# A high-quality dataset featuring classified and annotated cervical spine X-ray atlas

**DOI:** 10.1038/s41597-024-03383-0

**Published:** 2024-06-13

**Authors:** Yu Ran, Wanli Qin, Changlong Qin, Xiaobin Li, Yixing Liu, Lin Xu, Xiaohong Mu, Li Yan, Bei Wang, Yuxiang Dai, Jiang Chen, Dongran Han

**Affiliations:** 1 School of Life Sciences, Beijing University of Chinese Medicine, Beijing, 102488 China; 2 Department of Dermatology, Air Force Medical Center, Air Force Medical University, Beijing, 710000 China; 3 Department of Orthopedics and Traumatology, Qiannan Traditional Chinese Medicine Hospital, Guizhou, 558000 China; 4 Shenzhen Hospital of Beijing University of Chinese Medicine, Shenzhen, 518172 China; 5 School of Management, Beijing University of Chinese Medicine, Beijing, 102488 China; 6 Department of Orthopedics, Dongzhimen Hospital, Beijing University of Chinese Medicine, Beijing, 100700 China; 7 School of Humanities, Beijing University of Chinese Medicine, Beijing, 102488 China; 8 Longhua Hospital, Shanghai University of Traditional Chinese Medicine, Shanghai, 200032 China

**Keywords:** Orthopaedics, Spinal cord diseases

## Abstract

Recent research in computational imaging largely focuses on developing machine learning (ML) techniques for image recognition in the medical field, which requires large-scale and high-quality training datasets consisting of raw images and annotated images. However, suitable experimental datasets for cervical spine X-ray are scarce. We fill the gap by providing an open-access Cervical Spine X-ray Atlas (CSXA), which includes 4963 raw PNG images and 4963 annotated images with JSON format (JavaScript Object Notation). Every image in the CSXA is enriched with gender, age, pixel equivalent, asymptomatic and symptomatic classifications, cervical curvature categorization and 118 quantitative parameters. Subsequently, an efficient algorithm has developed to transform 23 keypoints in images into 77 quantitative parameters for cervical spine disease diagnosis and treatment. The algorithm’s development is intended to assist future researchers in repurposing annotated images for the advancement of machine learning techniques across various image recognition tasks. The CSXA and algorithm are open-access with the intention of aiding the research communities in experiment replication and advancing the field of medical imaging in cervical spine.

## Background & Summary

Cervical spine diseases are recognized as a public health issue, characterized by diversity and high morbidity, which contained mainly cervical spondylosis, malformations, fractures, instability, and spondylolysis^1,2^. Over a third of a billion people suffered from persistent mechanical neck pain for at least three months, as indicated by a global assessment in 2015^3^. X-ray is a common and cost-effective method to evaluate cervical spine diseases, especially in screening and follow-up^4,5^. It is imperative for post-operative assessment in Anterior Cervical Corpectomy and Fusion (ACCF), Anterior Cervical Discectomy and Fusion (ACDF), and Anterior Cervical Disc Replacement (ACDR)^6^.

Quantitative parameters in X-ray imaging serve as the critical content of assessment for cervical spine diseases^7^. In routine clinical practice, surgeons primarily rely on manual measurements or visual assessments, with disparities in professional expertise contributing to an elevated risk of misdiagnosis and measurement inaccuracies. The results of manual measurements are usually obtained by taking the average of measurements from multiple surgeons. Nevertheless, this time-consuming and labor-intensive method lacks cross-checking^8^. Thus, failing to reduce the subjective impact of surgeons and unable to mitigate inherent errors associated with manual measurements. Moreover, the vast array of quantitative parameters for cervical spine disease assessment are extremely difficult to be obtained by manual measurement.

Machine learning (ML) can assist and replace manual efforts in performing extensive and precise complex calculations. Nevertheless, ML requires large-scale and high-quality training datasets consisting of raw images and annotated images. Presently, the publicly accessible large X-ray datasets predominantly encompass chest radiographs and fractures, with a portion of the studies incorporating merely classification data, thus lacking annotations requisite for quantitative analysis^9–13^. Existing datasets of cervical spine X-rays, which amalgamate images of the cervical, thoracic, lumbar, and whole spine^14^, exhibit considerable variability stemming from the distinct anatomical structures of the vertebral body and their unique physiological and pathological characteristics. Such marked differences in data characteristics significantly limit their suitability for machine learning, as the heterogeneity hampers the consistent application required for effective algorithmic training. Additionally, previous datasets present problems with small sample size, inconsistent image clarity, or are primarily used for reclassification tasks based on existing datasets (instead of creating a new dataset). Evidently, suitable datasets for cervical spine X-ray are scarce. To fill the gap, we developed Cervical Spine X-ray Atlas (CSXA), a dataset specifically and meticulously designed for the application of ML in the realm of cervical spine imaging.

Ensuring the quality of image annotations is crucial for the integrity of the entire dataset. Image annotations play a key role not only in machine learning applications but also essential for algorithms focused on measuring quantitative parameters. These quantitative parameters are derived from the annotations of vertebral keypoints, including the four corner points and the centroid of each vertebra^15,16^. This allows us to compute quantitative parameters from the keypoints in image annotation. The annotation of keypoints is full of challenges in specific images of vertebral body ghosting, defects, artifacts, bone hyperplasia, and osteoporosis, which are retained in this dataset to generate a robust and generalizable ML model. Non-specialist orthopedic spine surgeons often struggle with accurate image annotation. Therefore, image annotation of keypoints was independently performed by four orthopedic spine surgeons with an average of 6 years of experience (range 3–12), and was cross checked three times.

The algorithm based on keypoints addresses the issues of laborious manual processes, measurement errors, lack of cross-checking, and incomplete parameters measurement^17^. However, quantitative parameters for diagnosing cervical spine diseases are actual distances, while algorithmic outputs are pixel values. A previous study adopted^16^ the ratio of distances within images due to the challenges in acquiring pixel equivalent. Pixel equivalent^18^, defined as the ratio of actual distance to pixel distance, plays a crucial role in converting a part of parameters in the study of cervical spine X-ray. It is essential to establish the relationship between pixel and physical dimensions to accurately translate these into actual distances and areas. In this study, we meticulously computed for each image with Python scripts by dividing the pixel values of the scale in each image by the corresponding graduated markings.

**Fig. 1:**
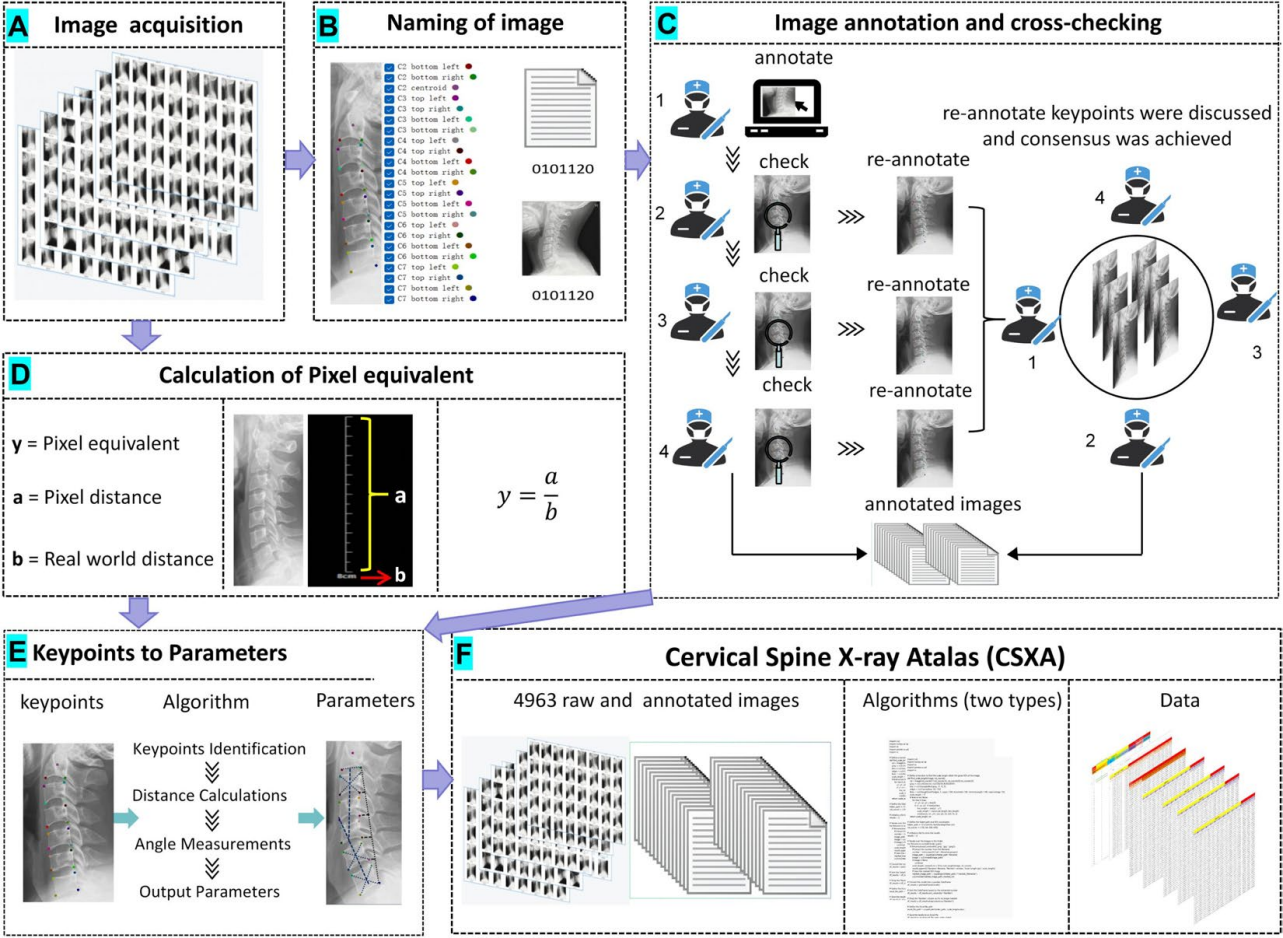
The flowchart for creating the CSXA dataset: (1) Image A illustrates the construction of the raw image of cervical spine X-ray. (2) Image B shows the naming of the keypoints, as well as the naming of the raw images and annotated images. (3) Image C depicts the methods of image annotation and cross-checking. (4) Image D is a schematic diagram illustrating the calculation of pixel equivalent. (5) Image E demonstrates the main algorithms used for converting annotated images into quantitative parameters. (6) Image F presents the complete data of the CSXA dataset, including 4963 raw and annotated images, two types of codes, and data about all basic information and quantitative parameters.

The CSXA, algorithm and basic information are open-access with the intention of aiding the research communities in experiment replication and advancing the field of medical imaging in cervical spine (Fig. 1).

## Methods

### Medical ethics

The ethics committee of Dongzhimen Hospital of Beijing University of Chinese Medicine approved this study (Ethical approval number: 2024DZMEC-126). Cervical spine X-rays removed any identifiable information except for gender and age, and other data underwent secondary processing based on these X-rays to protect patients’ privacy. We received an exemption from individual informed consent, as obtaining informed consent would hinder the study.

#### Image annotation

##### Image annotation tool

Image annotation of keypoints was independently performed by four orthopedic spine surgeons who had an average of 6 years of experience (range 3-12), and was cross checked three times. The cross-checking labels were referred to a senior orthopedic spine surgeon with over 12 years of experience for the final review and validation. All data were annotated using the labelme plug-in (pip install labelme) from Anaconda Powershell Prompt (Anaconda3) in ANACONDA (https://www.anaconda.com).

#### Selection of keypoints

Keypoints selection is foundational to subsequent analyses in cervical spine X-ray studies. Selected keypoints include the inferior endplate of C2, the central point of C2, and the corners of C3-C7 vertebrae, which are extensively used to generate diagnostic parameters, encompassing a wide range of lines and angles. The algorithm is designed for parameter calculations, characterized by its objectivity, reproducibility, and accuracy. It increases the number of keypoints and performs essential parameter calculations based on these keypoints. The upper endplate of the C2 (axis) vertebra and the C1 (atlas) were not selected due to their unique osseous connections and the absence of an intervertebral disc, which create unclear boundaries on X-rays. This method of keypoint selection and annotation is particularly well-suited for the quantitative analysis and classification of cervical spine diseases.

#### Naming of keypoints

**Fig. 2:**
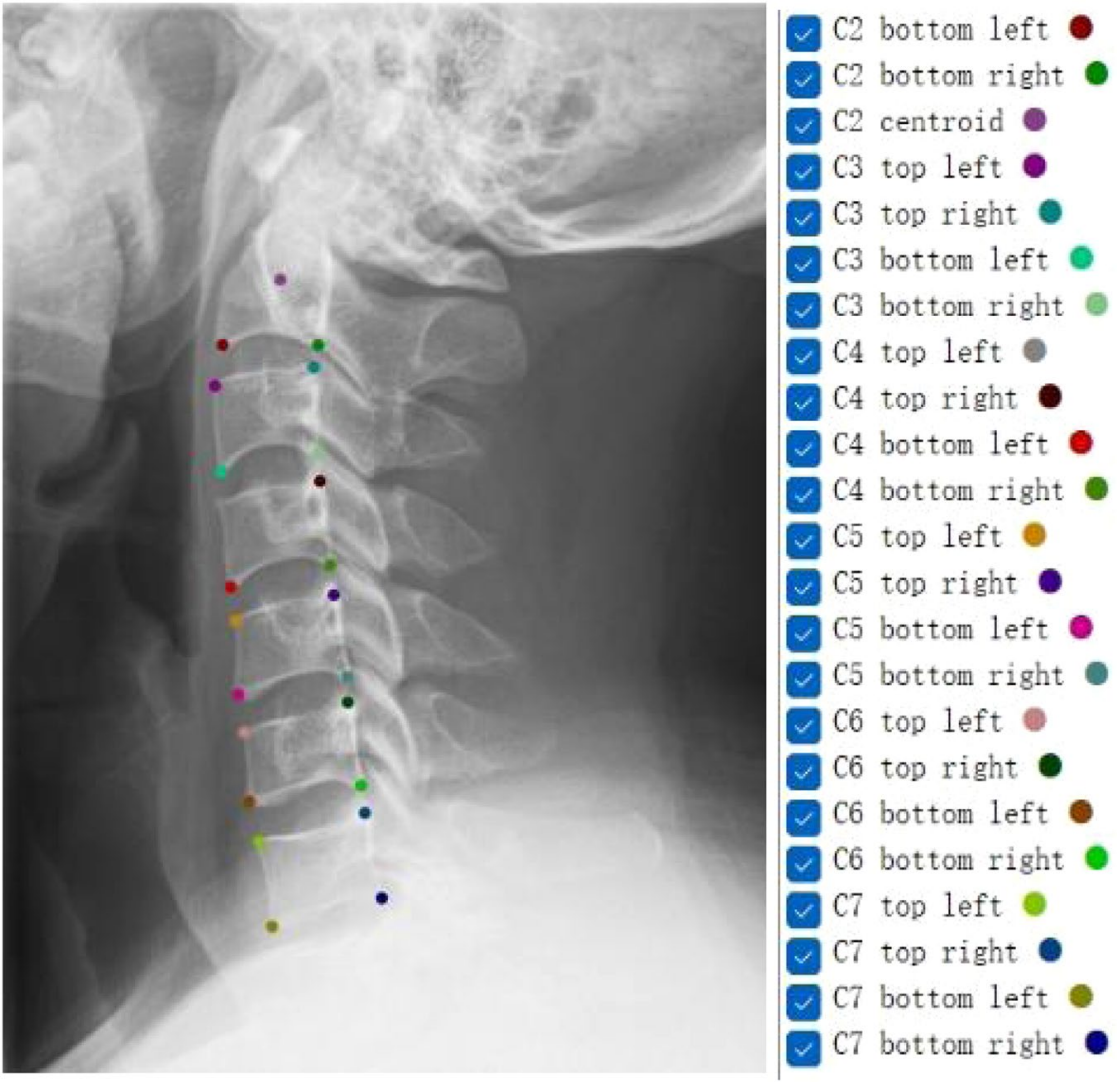
Fully tagged and labelled sample image.

Every annotated JSON image contains 23 keypoints, which are annotated using different colors by the labelme. The color and name of the keypoints of every image are consistent. The keypoints include the four corner points of the third to seventh cervical vertebral body (C3 to C7), and the central point and the two corner points of lower endplate of C2. The corner points are the four intersections which formed by the upper and lower endplates of the vertebral body with the anterior and posterior edges. The keypoints were named as indicated in the legend to Fig. 2.

#### Cross-checking of keypoints

Researcher 1, with three years of experience, initially annotated all 4,963 images. Researcher 2, also with three years of experience, reviewed the annotations and selected images requiring modifications, passing on the remaining, unselected images to Researcher 3. Researcher 3, With six years of experience, reviewed the transferred images and again selected those needing further modifications, forwarding the rest to Researcher 4. Researcher 4, with twelve years of experience, performed the final individual review, selected additional images for modifications. Finally, the images selected by Researchers 2, 3, and 4 as needing modifications were reviewed and discussed by all four researchers to finalize the amendments.This sequential and multi-tiered screening process effectively harnesses the expertise of different researchers and ensures the high quality of annotations.

#### Picture naming

**Fig. 3:**
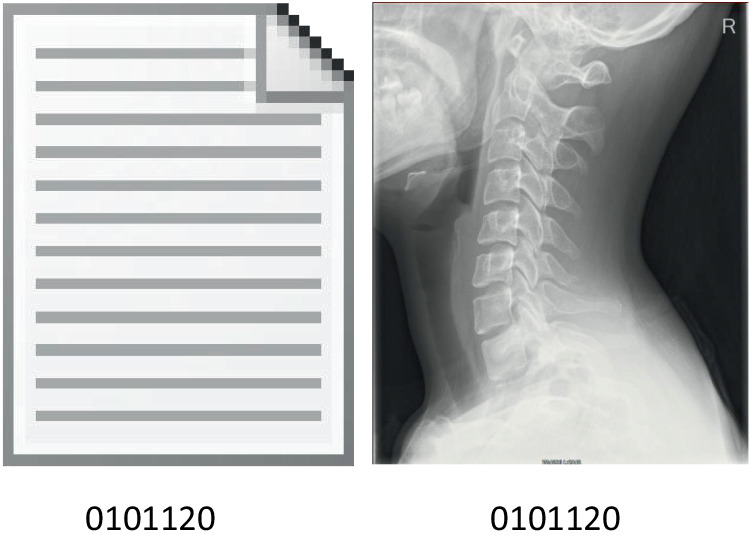
Schematic diagram of picture naming. ‘0101120’ denotes the 101 sample, a 20-year-old female.

The preceding four digits represent the image’s sequence number with a range from 0001 to 5000 in the CSXA, and the fifth digit is the gender code (1 for female, 0 for male). The final two digits are the age (ages 10 and above are represented directly; ages below 10 are indicated with a leading 0). The names of raw images are the same as the corresponding annotated JSON files (Fig. 3).

#### Population classification

The CSXA consists of two population groups: asymptomatic individuals and symptomatic patients. The inclusion of asymptomatic participants was from individuals undergoing health checkups for personal reasons at the International Department of Dongzhimen Hospital, affiliated with Beijing University of Chinese Medicine. Symptomatic persons are included individuals visiting at Dongzhimen Hospital, Beijing University of Chinese Medicine.

#### Doctor label of cervical curvature

According to the Modified Toyama^19^ cervical curvature classification, the cervical curvature is classified into four groups: Lordotic group, Straight group, Sigmoid group, and Kyphotic group. Interestingly, during our manual sorting of images, we discovered that the Sigmoid group in the Modified Toyama classification can be further divided into two main subtypes. We classified the posterior convexity of the C3 and C4 vertebral body as Sigmoid 1, and the posterior convexity of the C5 and C6 vertebral body as Sigmoid 2.

**Quantitative parameters based on keypoints data analysis:**

1. Disc height^20^: The vertical and straight-line distance between the corner points of two adjacent vertebral bodies (Fig. 4A,D);

**Fig. 4:**
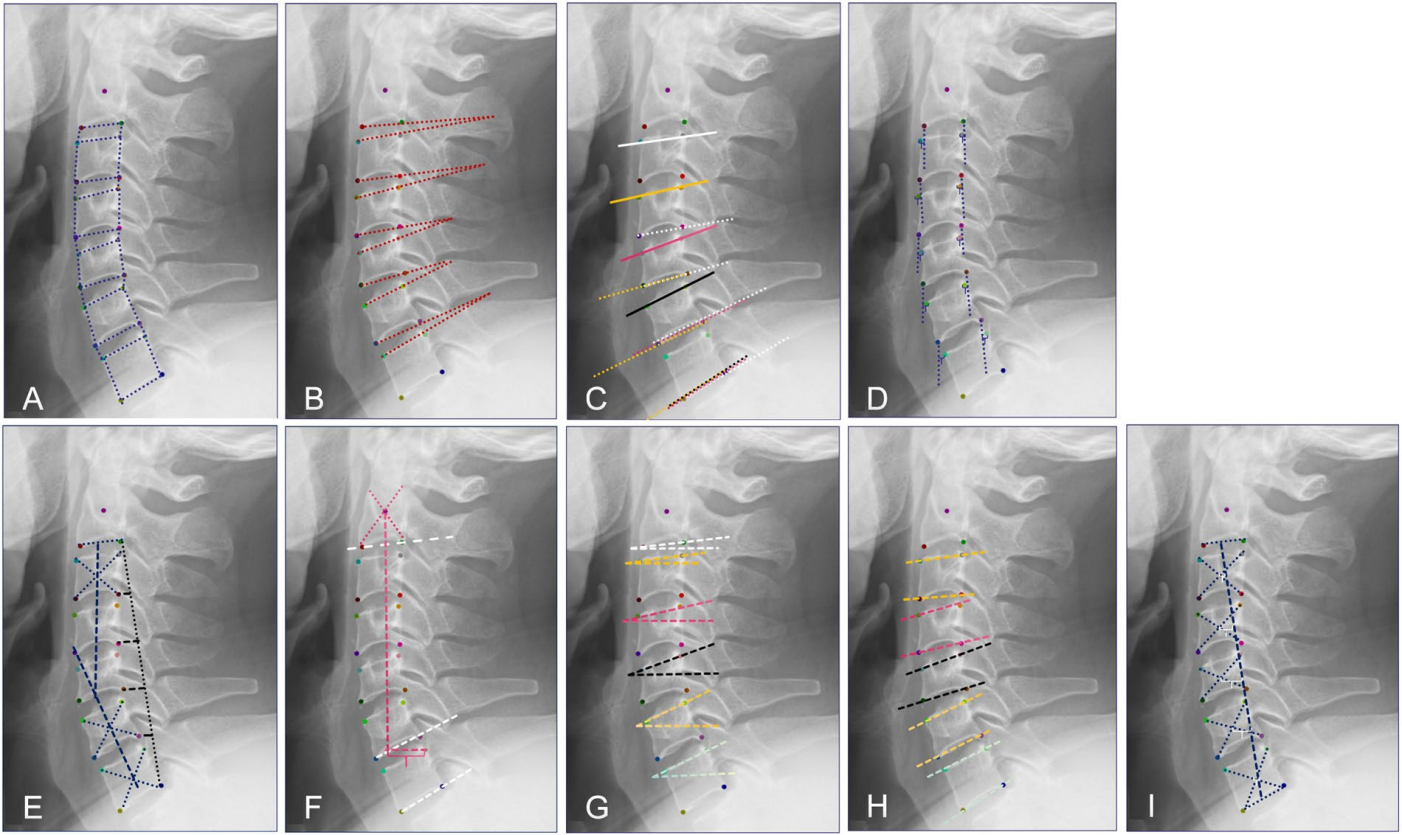
Schematic diagram of quantitative parameters.
2. Vertebral body^16^: The straight-line distances of the anterior and posterior sides, as well as between the superior and inferior endplates of a vertebral body, are measured between the corner points (Fig. 4A);
3. Cervical disc angle^21^ (CDA): The CDA was defined as the angle formed by the endplates of the upper and lower vertebral bodies (Fig. 4B). Furthermore, a classification with positive on the posterior side of the vertebral body and negative on the anterior side was provided to meet the needs of different research communities.
4. Functional Spinal Unit^22^ (FSU): FSU consists of an upper and a lower vertebra with an intact intervertebral disc (Fig. 4C), and a classification with positive on the posterior side of the vertebral body and negative on the anterior side.
5. Parameters of cervical spine instability^23^: Radiologic diagnosis of instability is the angle of adjacent vertebrae greater than 11 degrees or anterolisthesis greater than 3.5 mm of one vertebral body on another. In fact, the angle between adjacent vertebrae is CDA. The anterolisthesis is the anterior-posterior distance of the corner points of adjacent vertebral bodies on a horizontal line. If the upper vertebra is to the left of the lower vertebra, it is counted as a positive number, and if to the right, as a negative number; calculate separately for the anterior and posterior edges of the two adjacent vertebral bodies (Fig. 4D).
6. Vertebral body slope^24^: C2 slope is defined as the angle between a line parallel to the lower endplate of the C2 vertebra and the horizontal plane. C3, C4, C5, C6, and C7 slope are defined as the angle between a line parallel to the upper endplate and the horizontal plane of the C2, C3, C4, C5, C6, and C7 vertebra, respectively (Fig. 4G). Furthermore, a classification with positive on the kyphosis and negative on lordosis to meet the needs of different research communities.
7. Cervical curvature: C2-7 Cobb angle is measured from the inferior endplate of C2 to the inferior endplate of C7, C2-6 Cobb is measured from the inferior endplate of C2 to the inferior endplate of C6, and C2-7 SVA is centroid of C2 and the posterior superior aspect of C7^25^ (Fig. 4F). Toyama Curvature^19^: the AB line (in Fig. 4I) refers to the line connecting the midpoint of the lower endplate of the C2 vertebra to the midpoint of the upper endplate of the C7 vertebra. Based on the position and distance of the centroids relative to the AB line, the cervical spine can be categorized into the following groups: Lordotic group: All centroids are anterior to the AB line, and the distance between at least one centroid and the AB line is 2 mm or more; Straight group: The distance between the AB line and each centroid is less than 2 mm; Sigmoid group: Some centroids are anterior and some are posterior to the AB line, and the distance between the AB line and at least one centroid is 2 mm or more; Kyphotic group: All centroids are posterior to the AB line, and the distance between at least one centroid and the AB line is 2 mm or more. We further classified the posterior convexity of the C3 and C4 vertebral body as Sigmoid 1, and the posterior convexity of the C5 and C6 vertebral body as Sigmoid 2. The distance between the AB line and at least one centroid is 2 mm or more (Fig. 4I). Cervical Curvature Index^26^ (CCI) measures cervical curvature by determining the distance from the posteroinferior edge of the C3-C6 vertebral bodies to a straight line drawn from the posteroinferior edge of C2-C7 [CI = (A + B + C + D)/E × 100], A: Distance from the posteroinferior edge of C3 to the line, B: Distance from the posteroinferior edge of C4 to the line, C: Distance from the posteroinferior edge of C5 to the line, D: Distance from the posteroinferior edge of C6 to the line, E: Total distance from the posteroinferior edge of C2-C7 to the line (Fig. 4E). The Centroid Measurement of Cervical Lordosis (CCL) method^19^ refers to the angle formed between the line connecting the midpoint of the lower endplate of C2 to the centroid of C3, and the line connecting the centroids of C6 and C7. In previous study, this value was considered negative when the C2-C3 line was posterior to the C6-C7 line. However, this negative value actually represents a normal physiological curvature. Therefore, for consistency in the study of cervical lordosis, we have redefined the situation where the C2-C3 line is posterior to the C6-C7 line as a positive value (Fig. 4E,F,I).
8. Vertebral Angle^26^: The vertebral body angle is the angle between the upper and lower endplates, and a classification with positive on the posterior side of the vertebral body and negative on the anterior side was provided (Fig. 4H).

#### Algorithm

The objective of image keypoints annotation is to derive quantitative parameters for medical diagnostics and treatment. We have devised an advanced algorithm that transforms 23 image keypoints into 77 detailed quantitative parameters. This algorithm effectively combines all previously described calculation methods and formulas, facilitating an automated, efficient, and accurate keypoints-based computation. The algorithm initiates with the establishment of a ‘points_dict’, a foundational step in correlating key points within images to their respective numerical indices. It employs ‘cal_dist_adj_row’ and ‘cal_dis_adj_col’ for the precise calculation of distances between proximate points, whether arrayed in rows or columns. In the realm of angular measurements, the algorithm utilizes ‘cal_angle’, ‘cal_angle_adj’, and ‘cal_angle_not_adj’. Here, ‘cal_angle’ is responsible for computing the angular relationship in a quartet of points, while ‘cal_angle_adj’ and ‘cal_angle_not_adj’ systematically calculate angles between adjacent and non-adjacent points, respectively. Additionally, the algorithm encompasses functions such as ‘cal_sva’, ‘cal_c_type’, ‘cal_cns’, ‘cal_cn’, and ‘cal_toyama’ for the quantification of critical spinal parameters, including the sva and cobb angles. Integral to this framework are advanced auxiliary functions like ‘cal_intersection_points’, ‘point_position_relative_to_line’, and ‘cal_dist_point_line’, which are instrumental in executing sophisticated geometric computations. The final step of the algorithm employs the ‘cal_json_folder’ function, which methodically reads each JSON file, performs the necessary calculations, and compiles the results into an Excel file, thereby completing the synthesis of a comprehensive set of cervical spine metrics. The final output of the algorithm, consisting of 77 quantified parameters, is manually categorized to yield 118 ultimate results to meet the diverse requirements for the parameters.

#### Population distribution

The CSXA encompasses a total of 4963 individuals, consisting of 3202 females and 1761 males. The age distribution across the entire cohort ranged from 18 to 87 years, with a majority, aged between 20 and 70 years accounting for 4824 individuals. There are 4782 symptomatic patients with cervical pain or cervical spondylosis symptoms and 181 asymptomatic individuals. A detailed distribution of age and curvature can be found in Table 1.

**Table 1:** Age distribution of cervical spine curvature (Modified Toyama).

Age	Lordotic	Straight	Sigmoid1	Sigmoid2	Kyphotic	Total
18–20 (age)	9	10	4	4	6	33
20–30 (age)	401	496	65	214	190	1366
30–40 (age)	617	625	110	217	204	1773
40–50 (age)	298	296	40	131	66	831
50–60 (age)	222	169	49	56	19	515
60–70 (age)	166	100	35	35	15	351
70–80 (age)	46	12	12	5	2	77
80–90 (age)	6	4	3	2	2	17
Total	1765	1712	318	664	504	4963

#### Pixel equivalent

**Fig. 5:**
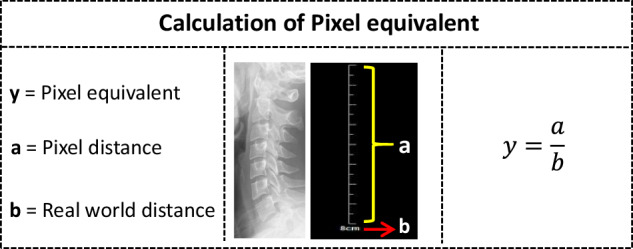
Schematic diagram of the calculation process for pixel equivalent.

Every raw image comes with a linear scale featuring distinct graduated markings of varying lengths and intervals, which allows us to convert the pixel distance between two pixels into the real-world distance by drawing a line along the scale of the image^17^. These graduated markings are meticulously recorded in an Excel spreadsheet for reference. Subsequently, a Python script is developed to uniformly compute the pixel distances of each scale within the images. Ultimately, the pixel equivalent is accurately calculated by dividing the pixel values of the scale in each image by the corresponding graduated markings (Fig. 5).

## Data Records

All demographic categorizations, spinal curvature data, gender, age, and pixel equivalent information have been recorded in the Excel file named “dataset”. This document, along with the code, is available on GitHub at https://github.com/yran888/CSXA-dataset.git. The entire image dataset has been stored in the ‘datasets’ folder, which includes two subfolders: ‘datasets-PNG’ and ‘datasets-JSON’. The folder has been uploaded to Science Data Bank^27^ and can be accessed at 10.57760/sciencedb.15391.

## Technical Validation

### Keypoints annotation validation

The annotations of the image underwent three meticulous cross-checking. Additionally, a Python script was developed to examine the count and nomenclature of all annotation points.

### Pixel value validation

Random sampling measurements were conducted using ImageJ (https://imagej.net/ij/download.html) to validate the consistency of results computed by python code.

### Quantitative parameter validation

Random cervical spine X-rays were sampled and measured using the PACS system for all quantitative parameters to verify the consistency between algorithm measurements and manual measurements.

### Results

The annotations for all keypoints positions, counts, and names were accurate, and the algorithm measurements aligned consistently with the manual measurements.

## Data Availability

The Python code used in this paper was developed in version 3.11.0 and is available for free access. The first code, ‘pixel equivalent’, is designed to calculate the pixel values of linear scales in images. The second code, which consists of ‘aux_info_cal’ and ‘test’, is used to calculate quantitative parameters.
